# Pandemic Influenza Infection Promotes Streptococcus pneumoniae Infiltration, Necrotic Damage, and Proteomic Remodeling in the Heart

**DOI:** 10.1128/mbio.03257-21

**Published:** 2022-01-04

**Authors:** Maryann P. Platt, Yi-Han Lin, Rosana Wiscovitch-Russo, Yanbao Yu, Norberto Gonzalez-Juarbe

**Affiliations:** a Infectious Diseases and Genomic Medicine Group, J Craig Venter Institute, Rockville, Maryland, USA; b Chemistry and Biochemistry Department, University of Delaware, Newark, Delaware, USA; Mississippi State University

**Keywords:** influenza, *Streptococcus pneumoniae*, proteomics, secondary bacterial infections, heart, necroptosis, adhesion, pneumolysin, pore-forming toxins

## Abstract

For over a century, it has been reported that primary influenza infection promotes the development of a lethal form of bacterial pulmonary disease. More recently, pneumonia events caused by both viruses and bacteria have been directly associated with cardiac damage. Importantly, it is not known whether viral-bacterial synergy extends to extrapulmonary organs such as the heart. Using label-free quantitative proteomics and molecular approaches, we report that primary infection with pandemic influenza A virus leads to increased Streptococcus pneumoniae translocation to the myocardium, leading to general biological alterations. We also observed that each infection alone led to proteomic changes in the heart, and these were exacerbated in the secondary bacterial infection (SBI) model. Gene ontology analysis of significantly upregulated proteins showed increased innate immune activity, oxidative processes, and changes to ion homeostasis during SBI. Immunoblots confirmed increased complement and antioxidant activity in addition to increased expression of angiotensin-converting enzyme 2. Using an *in vitro* model of sequential infection in human cardiomyocytes, we observed that influenza enhances S. pneumoniae cytotoxicity by promoting oxidative stress enhancing bacterial toxin-induced necrotic cell death. Influenza infection was found to increase receptors that promote bacterial adhesion, such as polymeric immunoglobulin receptor and fibronectin leucine-rich transmembrane protein 1 in cardiomyocytes. Finally, mice deficient in programmed necrosis (i.e., necroptosis) showed enhanced innate immune responses, decreased virus-associated pathways, and promotion of mitochondrial function upon SBI. The presented results provide the first *in vivo* evidence that influenza infection promotes S. pneumoniae infiltration, necrotic damage, and proteomic remodeling of the heart.

## OBSERVATION

For decades, extensive evidence has implicated risk of myocardial infarction in various infectious diseases such as influenza, pneumococcal pneumonia, and bacteremia (1–3). Our group has previously shown that the bacterium Streptococcus pneumoniae (pneumococcus) is capable of myocardial invasion, induction of cardiomyocyte death, and disruption of cardiac contractility and function during acute infection and convalescence (4–6). Cardiac complications have also been observed during influenza epidemics and pandemics. Influenza has been shown to disseminate from the lungs to cardiac tissue and cause myocarditis, ischemia, and cardiac dysfunction, among other adverse cardiac events (3, 7–11). Recently, our group reported that pandemic influenza A virus (IAV) can persist in the heart after clearance of pulmonary infection, remodeling the cardiac proteome and phosphoproteome (12).

While separately, IAV and S. pneumoniae cause millions of infections per year (https://www.who.int/news-room/fact-sheets/detail/pneumonia), it has been recognized that IAV promotes the development of a lethal form of pneumococcal pulmonary disease (13). For example, during the IAV 1918 pandemic, S. pneumoniae was recovered in ∼95% of all fatal cases, cementing the important role of co- and secondary bacterial infections (SBI) during pandemic events (14). Whereas progress has been made in understanding the basis for this deadly synergism (15), the molecular mechanisms and the extent of synergistic disease in other organs remain unknown. Importantly, it has recently been shown that necroptosis is a major driver of pulmonary damage during SBI (16). Necroptosis is driven by activation of the effector protein-mixed lineage kinase domain-like (MLKL), leading to disassociation of cellular membranes (17). The role of necroptosis in the heart upon SBI is not known.

Due to the synergistic nature of these pathogens in the pulmonary setting and their tropism toward the heart, we hypothesized that primary influenza infection leads to potentiation of S. pneumoniae cardio-pathological effects. Here, we use label-free quantitative proteomics and molecular approaches to define the synergistic effect of IAV and S. pneumoniae in hearts of mice using a model of SBI.

### Pandemic influenza virus infection promotes S. pneumoniae translocation to the heart.

**FIG 1:**
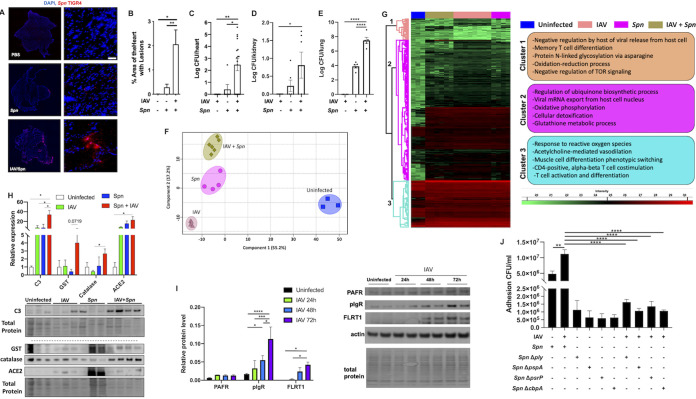
Primary pandemic influenza virus infection promotes S. pneumoniae translocation to the heart, upregulation of adhesion factors, and global proteome remodeling. Male and female 6- to 8-week-old C57BL/6N mice were intranasally infected with A/California/7/2009 (IAV) at day 0. At day 10, mice were infected with S. pneumoniae intratracheally at a dose of 1 × 10^3^ CFU. Mice were euthanized, and heart tissue was collected at day 12. (A) Immunofluorescent staining for S. pneumoniae capsule (red) in cardiac tissue sections. Cell nucleus was stained in blue. White bar, 50 μm. DAPI, 4′,6-diamidino-2-phenylindole. (B) Percentage of area of heart with lesions was measured using ImageJ. Bacterial titers (in log CFU) in homogenized hearts (C), kidneys (D), and lungs (E) upon euthanasia. (F and G) Principal-component analysis (PCA) (F) and hierarchical clustering (G) of LFQ intensities of significantly changed proteins (ANOVA, FDR of 0.05) among uninfected, IAV-infected, S. pneumoniae-infected, and IAV and S. pneumoniae-infected hearts. Enriched GO biological process terms are indicated for marked clusters. (H) Immunoblots for complement C3 (C3), catalase, GST, and ACE2. (I) Immunoblots for complement PAFr, pIgR, and FLRT1. Protein level quantification was performed using ImageJ. (J) Adhesion assay CFU/mL of cells infected with or without IAV at an MOI of 2 for 2 h and then challenged with S. pneumoniae WT, *Δply*, *ΔpspA*, *ΔpsrP*, or *ΔcbpA.* Proteomic data are representative from 2 separate experiments done with 3 mice of each sex; no sex-based differences were observed. Kruskal-Wallis test with Dunn’s multiple-comparison posttest was performed. Asterisks denote the level of significance observed as follows: *, *P* ≤ 0.05; **, *P* ≤ 0.01; ***, *P* ≤ 0.001; and ****, *P* ≤ 0.0001.

Male and female wild-type C57BL/6 mice were infected with mouse-adapted pandemic IAV (strain A/California/7/09) (12) for 10 days. At this time point, viral titers are undetectable in the lungs, as has been previously described by our group (12, 16). Mice were then challenged with S. pneumoniae (strain TIGR4) at a dose of 1 × 10^3^ CFU for 2 days before euthanasia. Immunofluorescent staining against S. pneumoniae showed cardiac microlesion (4) formation only in animals preinfected with IAV (Fig. 1A), with a significant increase in the percentage of area of heart with lesions (Fig. 1B). Bacterial titers in the heart were almost 1,000 times higher in SBI than mice infected with pneumococcus alone (Fig. 1C). Without IAV preinfection, mice are required to be infected with a dose of 1 × 10^6^ CFU to achieve comparable S. pneumoniae cardiac translocation to that observed in Fig. 1A to C (5, 6, 18, 19). Of note, bacterial titers in the kidneys and lungs of mice also showed a significant increase during SBI (Fig. 1D and E).

### Secondary bacterial infection leads to cardiac proteome remodeling.

We used high-resolution accurate liquid chromatography-tandem mass spectrometry (LC-MS/MS) platform and the label-free quantitation (LFQ) approach to assess cardiac proteomic changes induced by influenza or S. pneumoniae infections and after SBI. The global proteome level assessed 1,269 total quantifiable proteins. Analysis of variance (ANOVA) and permutation-based false-discovery rate (FDR) of 0.05 resulted in 288 proteins that showed variation among the four experimental conditions. Principal-component analysis (PCA) clustered biological replicates, suggesting differential proteomic profiles for each group (Fig. 1F). Single-infection groups clustered separately, implying distinct changes in cardiac host responses. The proteome of mice hearts experiencing SBI did not cluster together with either single infection, suggesting further proteome remodeling (Fig. 1F). Based on abundance profiles, proteins could be classified into three distinct clusters visualized in a heatmap (Fig. 1G; Data File S1 in the supplemental material). Gene ontology (GO) enrichment analysis of biological processes using proteins in cluster 1 revealed upregulation of terms associated with regulation by host of viral release from host cell, T-cell differentiation, and oxidation-reduction processes, among others. Clusters 2 and 3 showed further upregulation of immune responses, oxidation, and metabolic processes (Fig. 1G). In contrast to cardiac pneumococcal translocation during invasive pneumococcal disease, immune responses were active (Fig. 1G) (5, 6, 18, 20), suggesting that antibacterial immune responses in the heart may be altered by prior exposure to IAV.

10.1128/mBio.03257-21.1 DATA SET S1 Global proteome changes in the heart of mice during secondary bacterial infection to influenza. Download Data Set S1, XLSX file, 0.2 MB. Copyright © 2022 Platt et al. 2022 Platt et al. https://creativecommons.org/licenses/by/4.0/ This content is distributed under the terms of the Creative Commons Attribution 4.0 International license.

Several significantly changed proteins were validated by immunoblot. Levels of glutathione transferase and catalase were observed to be most increased in the secondary infection group (Fig. 1H). Complement component C3 and angiotensin I-converting enzyme 2 (ACE2) were observed upregulated in all infection conditions (Fig. 1H). C3 increase in all infection conditions suggests this ancient and conserved innate immune effector may be essential for cardiac responses against invading pathogens (12, 19). ACE2 has been shown to have a key role in pulmonary injury during influenza infections and is the primary receptor for SARS-CoV-2 (19).

### Primary influenza infection promotes pneumococcal adhesion in cardiomyocytes.

A major mechanism as to how IAV infection leads to increased pathogenesis of S. pneumoniae is the upregulation of host adhesion factors in the pulmonary setting (15). To test if IAV drives upregulation of adhesion factors in cardiomyocytes, we infected human AC16 cardiomyocytes with IAV (multiplicity of infection [MOI] of 2) for 24, 48, or 72 h, and expression of platelet-activating factor receptor (PAFr), polymeric immunoglobulin receptor (pIgR), and fibronectin leucine-rich transmembrane protein 1 (FLRT1) was measured by immunoblots (Fig. 1I). Both pIgR and FLRT1 were found to be upregulated upon IAV infection. We then used an *in vitro* model of sequential coinfection in AC16 cardiomyocytes, and cells were infected with IAV for 2 h (MOI of 2) and subsequently infected with S. pneumoniae wild type (WT) or mutants deficient in pneumolysin (*Δply*), pneumococcal surface protein A (*ΔpspA*), pneumococcal serine-rich repeat protein (*ΔpsrP*), and choline-binding protein A (*ΔcbpA*) for 4 h (MOI of 10). All mutants showed reduced adhesion in cells preinfected with IAV, while the WT strain showed increased adhesion (Fig. 1J). Together, the presented data provide a mechanism as to how IAV can enhance S. pneumoniae cardiac tropism via upregulation of bacterial adhesion factors.

### Influenza infection primes cardiomyocytes to pneumococcus-induced necroptosis.

**FIG 2:**
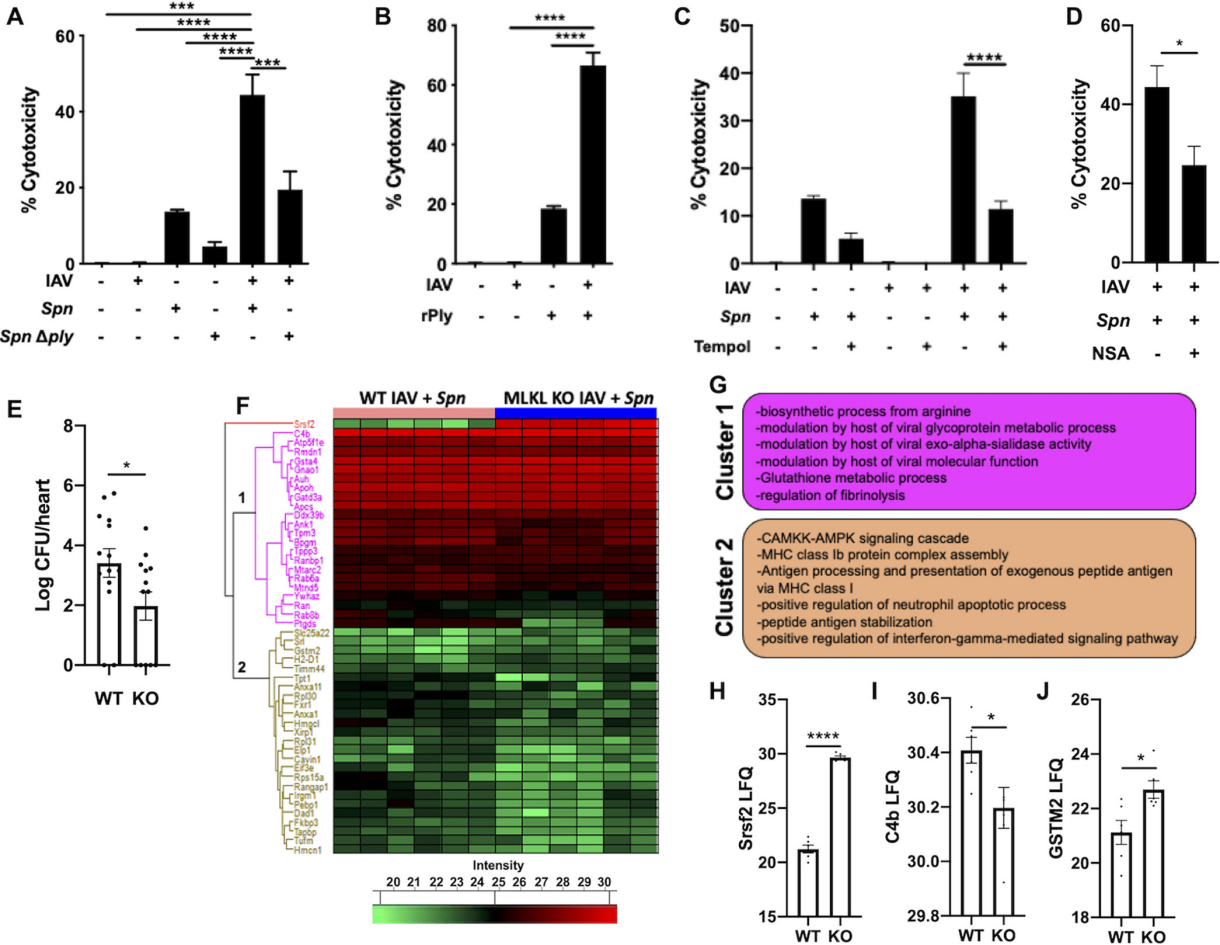
Influenza virus infection potentiates pneumococcus-induced cardiomyocyte toxicity. (A and B) Cytotoxicity levels in AC16 cells infected with H1N1 at an MOI of 2 for 2 h and then challenged with S. pneumoniae or S. pneumoniae *Δply* at an MOI of 10 (A) or challenged with recombinant pneumolysin (0.3 μg) for 4 additional hours (B). (C and D) Cytotoxicity of cells after pretreatment for 1 h with superoxide dismutase mimetic, Tempol (10 μM) (C), or necrosulfonamide (10 μM) (D) and subsequently infected as described above. (E) Bacterial titers (in log CFU) in homogenized hearts of IAV/S. pneumoniae-infected hearts of WT C57BL/6 and MLKL KO mice upon euthanasia. (F) Hierarchical clustering of LFQ intensities of significantly changed proteins (ANOVA, FDR of 0.05) among IAV/S. pneumoniae-infected hearts of WT C57BL/6 and MLKL KO mice. (G) Enriched GO biological process terms are indicated for marked clusters. (H to J) Histograms of label-free quantitation-based intensity of Srsf2 (H), C4b (I), and GSTM2 (J). Student's *t* test or Kruskal-Wallis test with Dunn’s multiple-comparison posttest was performed. Asterisks denote the level of significance observed as follows: *, *P* ≤ 0.05; **, *P* ≤ 0.01; and ***, *P* ≤ 0.001.

Primary influenza infection leads to potentiation of bacteria-mediated necrosis in the lungs in a ply-dependent manner (21, 22). We first observed potentiation of cellular toxicity when cardiomyocytes were initially infected with IAV *in vitro* (Fig. 2A). Of note, observed toxicity was partially dependent on ply activity, as *Δply* failed to show a synergistic effect (Fig. 2A). This was further supported by challenge of cardiomyocytes with recombinant pneumolysin after IAV infection (Fig. 2B). These results suggest ply can be targeted to reduce S. pneumoniae-driven cardiac injury during SBI. Proteomic data of IAV-infected hearts showed differential changes in proteins associated with oxidative stress (Fig. 1G and H; Data File S1). We hypothesized that such stress may influence cytotoxicity of cardiomyocytes upon S. pneumoniae infection. Pretreatment of cardiomyocytes with the general antioxidant Tempol (12) significantly reduced synergistic cardiomyocyte toxicity (Fig. 2C). Similarly, we observed that synergistic cytotoxicity was reduced by the selective inhibitor of MLKL, necrosulfonamide (Fig. 2D). Proteome analysis of hearts of WT and MLKL-deficient (MLKL knockout [KO]) mice experiencing SBI showed significant changes in 48 proteins (Fig. 2E; Data File S2). GO terms associated with major clusters showed decreases in viral-host interactions, coagulation, and glutathione metabolism and an increase in immune activity in MLKL KO during SBI (Fig. 2F). The most significantly changed protein was found to be Srsf2, a member of the serine/arginine family of pre-mRNA-splicing factors. RNA viruses have been shown to alter the function of Srsf2 to promote viral replication (23), suggesting MLKL KO animals may have an enhanced antiviral response. MLKL deficiency also led to reduced complement and an increase in antioxidant activity (Fig. 2G to I).

10.1128/mBio.03257-21.2 DATA SET S2 Comparison of proteome changes in hearts of wild-type mice versus MLKL-deficient mice experiencing SBI. Download Data Set S2, XLSX file, 0.02 MB. Copyright © 2022 Platt et al. 2022 Platt et al. https://creativecommons.org/licenses/by/4.0/ This content is distributed under the terms of the Creative Commons Attribution 4.0 International license.

In summary, our results indicate that influenza virus and S. pneumoniae each induce proteomic remodeling of the heart, and this is further exacerbated during SBI, leading to enhanced toxicity, necrotic cell death, and major proteomic changes. Taken together, our data show new evidence as to how pulmonary pathogens synergize to promote pathogenesis in extrapulmonary organs.

### Ethics statement.

Animal experiments were performed using protocols approved by the Institutional Animal Care and Use Committee at The University of Alabama at Birmingham and The University of Maryland College Park. Animal care and protocols followed the NIH Guide for the Care and Use of Laboratory Animals.

### Mouse models and influenza infection.

Male and female 6-week-old C57BL/6N (B6NTac) mice were obtained from Taconic Biosciences (Rensselaer, New York). MLKL KO mice were obtained through a material transfer agreement (MTA) with Warren Alexander (Walter and Eliza Hall Institute of Medical Research Parkville, Victoria, Australia) (24). Mice were intranasally challenged with 250 PFU of pandemic influenza virus A/California/7/2009. At day 10, mice were infected with 1 × 10^3^ of S. pneumoniae TIGR4 intratracheally as previously described (16). At 12 days postinfluenza infection, mice were sacrificed for cardiac tissue collection. The data presented are representative of two separate experiments totaling 6 mice, 3 of each sex.

### Mouse heart collection, protein digestion, and LC-MS/MS.

Mouse hearts were dissected, washed with cold phosphate-buffered saline (PBS) (3 times), snap frozen, and stored at −80°C. Protein extraction was performed using 2× SED lysis buffer (4% SDS, 50 mM EDTA, 20 mM dithiothreitol [DTT], 2% Tween 20, and 100 mM Tris-HCl, pH 8.0). The proteins were digested following the suspension trapping (STrap) protocol with self-packed glass fiber filters. The peptides were desalted using C_18_-based StageTip and stored at −80°C until further use. The LC-MS/MS analysis was carried out using an UltiMate 3000 nano-LC system coupled to Q Exactive mass spectrometer (Thermo Scientific). Peptides were first loaded onto a trap column (PepMap C_18_; 2 cm by 100 μm; Thermo Scientific) and then separated by an in-house-packed analytical column (C_18_ ReproSil; 3.0 mm; 20 cm by 75 μm inside diameter [i.d.]; Maisch GmbH) using a binary buffer system (buffer A, 0.1% formic acid in water; buffer B, 0.1% formic acid in acetonitrile) with a 150-min gradient (2 to 35% buffer B over 105 min, 35 to 80% buffer B over 10 min, back to 2% B in 5 min for equilibration after staying on 80% B for 5 min). MS data were acquired in a data-dependent top 10 method with a maximum injection time of 20 ms, a scan range of 350 to 1,800 Da, and an automatic gain control (AGC) target of 1e6. MS/MS was performed via higher-energy collisional dissociation fragmentation with a target value of 5e5 and maximum injection time of 100 ms. Full MS and MS/MS scans were acquired at resolutions of 70,000 and 17,500, both at *m/z* 200, respectively. Dynamic exclusion was set to 20 s.

### Proteome quantitation and bioinformatics analysis.

Protein identification and quantitation were performed using the MaxQuant-Andromeda software suite (version 1.6.5.0) with default parameters. A mouse protein database (17,040 UniProt sequences; reviewed only; version 02/2019) was used for the database search. For global proteome analysis, the following parameters were applied: 4.5-ppm and 20-ppm mass tolerances for precursor and fragments, respectively; trypsin as enzyme and allowance of maximally two missed cleavage sites; protein N-terminal acetylation and methionine oxidation as variable modifications; cysteine carbamidomethylation as a fixed modification; and peptide length is at least 7 amino acids. For global quantitative proteome analysis, the technical replicates (e.g., different LC-MS runs) were merged during MaxQuant analysis. The output result was first filtered to exclude “only identified by site,” “potential contaminant,” and “reversed” hits and then log_2_ transformed. For ANOVA analysis, the proteins were first filtered to include those that were quantified in at least two of the total three biological replicates of one condition. Gene ontology and KEGG pathway analyses were performed using DAVID Bioinformatics Resources 6.8 (https://david.ncifcrf.gov/home.jsp).

### Western blotting.

Samples for Western blotting were homogenized in PBS and sonicated and then diluted in Laemmli buffer and aliquoted for storage at −80°C. Samples were loaded into 4 to 15% gradient gels at 10 μg per lane, separated by SDS-PAGE, and transferred to nitrocellulose membranes. Total protein was quantified by Ponceau stain, and then membranes were blocked in blocking buffer (Tris-buffered saline [TBS]-0.01% Tween 20 containing 5% bovine serum albumin [BSA]) for at least 1 h. Primary antibodies were diluted in blocking buffer, and membranes were incubated overnight at 4°C. Primary antibodies used included glutathione *S*-transferase (GST) (1:1,000; Cell Signaling; catalog no. 2622), catalase (1:1,000; ProteinTech; catalog no. 21260-1-AP), ACE2 (1:1,000; ProteinTech; catalog no. 21115-1-AP), and C3 (1:1,000, ProteinTech; catalog no. 21337-1-AP). Horseradish peroxidase (HRP)-tagged secondary antibodies were used to detect primary antibodies (1:10,000 in blocking buffer) for 1 h. SuperSignal West Pico Plus (Thermo Fisher; catalog no. 34580) was used to develop HRP. All images were collected on an Amersham Imager 680 (GE) and analyzed for densitometry in ImageJ.

### Immunofluorescence.

Hearts were embedded in OCT and flash frozen for sectioning on a Thermo CryoStar NX50 cryostat. Hearts were sectioned onto glass slides at 6 mm. For staining, sections were air-dried and then fixed in ethanol/acetone and washed with PBS containing 0.1% Triton X-100 (PBST). Sections were then blocked for at least 1 h in 10% BSA in PBST. Sections were incubated overnight at 4°C in the following primary antibodies diluted in 1% BSA-PBST: type 4 SSI pneumococcus type antisera (1:1,000; Cedarlane, catalog no. 16747[SS]). Primary antibodies were detected with secondary antibodies conjugated to Alexa Fluor 647. Following secondary antibody incubation, slides were treated with NucBlue to label nuclei (1 drop per 1 mL) and covered with a coverslip. Images were acquired using a Nikon microscope with a 40× oil immersion lens. Images were analyzed in ImageJ.

### Cells and infections.

AC16 human cardiomyocytes (MilliporeSigma, Darmstadt, Germany) were grown to the multinucleated myotube phenotype and then infected with pandemic influenza A/California/7/2009 (H1N1) at an MOI of 2 for 2 h. Then cells were infected with wild-type S. pneumoniae (strain TIGR4), *Δply*, pneumococcal surface protein A (*ΔpspA*), pneumococcal serine-rich repeat protein (*ΔpsrP*), and choline-binding protein A (*ΔcbpA*) (all in TIGR4 background) at an MOI of 10 or challenged with recombinant ply for 4 h. Cells that received an inhibitor targeting reactive oxygen species (ROS) or MLKL were pretreated with the inhibitor Tempol at 10 μM or necrosulfonamide at 10 μM (Sigma-Aldrich, St. Louis, MO) for 1 h before infection and continued for the duration of the experiment. For *in vitro* studies, data from ≥3 separate experiments are shown.

### Statistical analyses for cell-based assays.

For multiple-group analyses, we used a nonparametric Kruskal-Wallis *H* test with Dunn’s *post hoc* analysis; grouped analyses were performed using a two-way ANOVA with Sidak’s *post hoc* analysis. These statistical analyses were calculated using Prism 7 (GraphPad Software, La Jolla, CA); *, *P* ≤ 0.05; **, *P* ≤ 0.01; ***, *P* ≤ 0.001.

### Data availability.

We declare that all data supporting the findings of this study are available within the manuscript and from the corresponding author upon reasonable request. Original proteomic data have been uploaded to ProteomeXchange with accession no. PXD016137.
